# Pediatric Vaccine Hesitancy in the United States—The Growing Problem and Strategies for Management Including Motivational Interviewing

**DOI:** 10.3390/vaccines13020115

**Published:** 2025-01-24

**Authors:** Ashlesha Kaushik, Julia Fomicheva, Nathan Boonstra, Elizabeth Faber, Sandeep Gupta, Helen Kest

**Affiliations:** 1 Division of Pediatric Infectious Diseases, Unity Point Health at St. Luke’s Regional Medical Center, University of Iowa Carver College of Medicine, 2720 Stone Park Blvd, Sioux City, IA 51104, USA; 2 Division of Clinical Psychology, Cambridge Health Alliance (CHA), Harvard Medical School, 1493 Cambridge Street, Cambridge, MA 02139, USA; jfomicheva@challiance.org; 3 Division of General Pediatrics, Blank Children’s Hospital, 1215 Pleasant Street, Des Moines, IA 50309, USA; nathan.boonstra@unitypoint.org; 4 Iowa Immunizes Coalition and Iowa Public Health Association, 501 SW 7th Street, Ste G, Des Moines, IA 50309, USA; elizabeth@iowapha.org; 5 Division of Pulmonary and Critical Care, Unity Point Health at St. Luke’s Regional Medical Center, 2720 Stone Park Blvd, Sioux City, IA 51104, USA; sandeep.gupta@unitypoint.org; 6 Division of Pediatric Infectious Diseases, St. Joseph’s Children’s Hospital, 703 Main Street, Paterson, NJ 07503, USA; kesth@sjhmc.org

**Keywords:** vaccine hesitancy, vaccine acceptance, strategies, policy, motivational interviewing

## Abstract

Vaccine hesitancy is a significant global issue and is recognized by the World Health Organization (WHO) as one of the most pressing threats to public health. Defined as the delay in acceptance or refusal of vaccines despite their availability, vaccine hesitancy undermines decades of progress in preventing vaccine-preventable diseases. The issue is complex, influenced by misinformation, distrust in healthcare systems, cultural beliefs, and access barriers. These challenges require innovative and empathetic solutions to increase vaccine acceptance. Addressing this growing epidemic requires a multifaceted approach, which involves broader strategies and policymaking and in addition, effective communication tools for clinicians. Motivational Interviewing (MI), a patient-centered communication technique, offers an effective strategy to address pediatric vaccine hesitancy by fostering trust, understanding, and informed decision-making. This review aims to explore the problem of pediatric vaccine hesitancy in the United States, examine its underlying factors, and highlight evidence-based strategies, including Motivational Interviewing, to address this growing concern in clinical and public health settings. It offers practical guidance for healthcare providers and pediatricians to tackle this growing problem effectively and emphasizes the need for a combined effort of communication, community outreach, education, and systemic policy to overcome vaccine hesitancy.

## 1. Introduction

The problem of vaccine hesitancy has become increasingly prevalent over the past decade, posing a significant challenge to public health, and has been named among the top 10 public health threats by the World Health Organization (WHO) [1]. Despite the proven safety and efficacy of vaccines in preventing life-threatening diseases, a growing number of individuals and communities delay or refuse immunizations. This hesitation undermines decades of progress in combating vaccine-preventable diseases in children and creates gaps in herd immunity, increasing the risk of outbreaks.

Vaccine hesitancy is not a singular issue but a complex phenomenon influenced by a combination of social, cultural, psychological, and systemic factors [2,3]. From historical mistrust in medical institutions to the widespread dissemination of misinformation through social media, the drivers of pediatric vaccine hesitancy are multifaceted and require nuanced approaches to address. Furthermore, the COVID-19 pandemic has amplified vaccine skepticism, with polarizing debates surrounding vaccine development timelines, safety, and mandates.

Despite the existence of safe and effective vaccines, a significant segment of the population remains hesitant or outright refuses vaccination. Common reasons for reluctance include fears about vaccine safety, perceived side effects, misinformation, and a lack of trust in healthcare systems [2,3]. This hesitancy not only thwarts efforts to reach herd immunity but also puts communities at risk of vaccine-preventable disease outbreaks, jeopardizing public health objectives.

In the U.S., pediatric vaccine exemptions have been steadily rising, leading to decreasing pediatric vaccination coverage rates [4]. Vaccine hesitancy poses significant challenges to achieving herd immunity and protecting vulnerable populations. Addressing these concerns requires advanced strategies that go beyond simply presenting scientific facts. Motivational Interviewing (MI) is an example of a patient-centered communication approach that can help alleviate concerns about vaccinations. Research shows that trust-building and patient-centered approaches in communication between pediatric providers and parents, such as Motivational Interviewing (MI), are essential for effectively engaging hesitant individuals [5,6]. By creating a supportive dialog, MI can help bridge the gap between vaccine hesitancy and vaccine acceptance.

Understanding the root causes of pediatric vaccine hesitancy and developing effective strategies to counteract it are critical for safeguarding public health. This review explores the problem and the causes and impact of pediatric vaccine hesitancy in the United States, highlights strategies implemented at various national and state levels, and describes MI techniques in detail to combat vaccine hesitancy.

### 1.1. Vaccine Hesitancy: The Problem

Vaccine hesitancy, defined by the World Health Organization (WHO) as the delay in acceptance or refusal of safe vaccines despite their availability, poses a significant public health challenge [1]. While a small percentage of Americans completely refuse all vaccines for their children, a more common behavior is delaying or spacing out routinely recommended childhood vaccinations, which still contributes to an overall decline in vaccine coverage [7]. Although vaccine coverage remains generally high, hesitancy is widespread. The U.S. Centers for Disease Control and Prevention’s (CDC’s) National Immunization Survey found that approximately one in five children had parents who reported vaccine hesitancy between 2019 and 2022 [8].

### 1.2. Causes of Vaccine Hesitancy

Vaccine hesitancy is driven by a complex interplay of misinformation, distrust in governmental and scientific authorities, cultural beliefs, and access issues. The reasons behind vaccine hesitancy vary by time, location, and the specific vaccine [2]. To better understand these complexities, the SAGE Working Group on Vaccine Hesitancy, an advisory group to the WHO, introduced the “3 Cs” model in 2015 [2]. This model categorizes determinants of hesitancy into the following:

1. Confidence: Trust in vaccine safety and efficacy, which can be undermined by misinformation, disinformation, and historical unethical practices in medicine.
2. Complacency: The perception that vaccine-preventable diseases pose minimal risk, particularly in populations with little to no experience with such illnesses due to successful vaccination programs.
3. Convenience: Barriers to vaccine access, such as affordability, availability, and logistical challenges.

Additional factors, including communication (how vaccine information is disseminated) and community influence (the role of community systems in shaping vaccine perceptions), further complicate vaccine hesitancy [9]. Within these domains, specific causes of vaccine hesitancy are numerous and varied. In the United States, pediatric vaccine hesitancy is associated with various social and political factors. For instance, vaccine hesitancy has been linked to social views like alternative health beliefs, as well as low perception of the risk of vaccine-preventable diseases [10]. Some parents might be “unsure” about vaccine efficacy and some might be fearful about the “large number of vaccine antigens” injected into the infant’s system. Vaccine hesitancy has also been found to be associated with socioeconomic factors like lower income and lower levels of education [11].

Notably, social media and the internet are likely factors in the rise of vaccine hesitancy in recent years. The amount of negative vaccine information on the internet and social media, and its impact on vaccine decision-making, has been extensively studied and discussed [12]. Parents who have more vaccine-hesitant contacts in their social networks are less likely to vaccinate [13]. Political polarization has been linked to the politicalization of vaccines in the U.S. and has fueled vaccine hesitancy, especially for COVID-19 vaccines during the pandemic [14]. Though few studies examined political affiliation and vaccine hesitancy prior to the COVID-19 pandemic, a 2018 study of conservative participants indicated a lower likelihood of immunization against measles, pertussis, and influenza [15]. Political affiliation has been strongly tied to COVID-19 vaccination hesitancy [16], but studies have not found consistent evidence that public polarization over the COVID-19 vaccine has led to increased measles, mumps, rubella (MMR) vaccine or routine infant vaccine refusal [17].

### 1.3. Impact of Vaccine Hesitancy

The consequences of vaccine hesitancy are far-reaching, posing an urgent threat to population health. By delaying or refusing vaccination, individuals not only place themselves at risk but also undermine herd immunity, increasing the likelihood of disease outbreaks, particularly among vulnerable populations such as infants, and immunocompromised individuals. This has been particularly evident with COVID-19, where unvaccinated individuals have been shown to be at a higher risk of hospitalization and need for critical care [18,19]. 

Nonmedical exemptions for school vaccinations have steadily increased over recent decades in the United States [4,20,21], highlighting a troubling trend. In the 2022–2023 school year, nonmedical exemptions in the U.S. reached a historic high of 3.0%, up from 2.6% the previous year [21]. This trend is particularly concerning in communities where vaccine refusals cluster, leading to significantly low immunization rates and heightened risks of outbreaks [22].

The consequences of pediatric vaccine hesitancy are evident in the resurgence of vaccine-preventable diseases. Measles, the most contagious vaccine-preventable disease, is particularly sensitive to immunization gaps. Between 2010 and 2019, the United States saw rising measles cases, including a widely publicized outbreak linked to Disneyland in 2014, and by 2019, measles cases reached their highest level in 25 years [23]. Although fewer cases were reported during the COVID-19 pandemic, 2024 has seen a resurgence (with more than 17-fold increase in cases in 2024 first quarter compared to the first quarter of 2020–2023) [24], underscoring the ongoing impact of vaccine hesitancy.

Parental vaccine refusal has also been linked to increased risks of other diseases, including pertussis, varicella, and invasive pneumococcal disease [25,26,27]. Nonmedical school exemptions have been associated with a higher incidence of pertussis and measles [28]. The persistence of vaccine hesitancy threatens public health efforts to control these diseases leading to outbreaks [29,30], increasing the burden of illness, hospitalizations, and preventable deaths.

Beyond its health implications, vaccine hesitancy carries significant economic costs. From 1994 to 2014, routine childhood immunizations in the United States prevented 508 million illnesses, 32 million hospitalizations, and 1,129,000 deaths, generating $540 billion in direct savings and $2.7 trillion in societal savings [31]. However, even modest declines in vaccination rates can erode these gains. For example, a 5% decrease in measles vaccine coverage could triple annual cases among children aged 2 to 11 years, costing the public sector an additional $2.1 million annually [32].

The WHO identified vaccine hesitancy as one of the top 10 health threats in 2019 [1], emphasizing the fact that mitigating vaccine hesitancy is not merely a public health priority but an urgent imperative. Without decisive action, its ongoing impact will lead to further disease outbreaks, strain healthcare systems, and erode the hard-won gains of vaccination programs. Urgent action is essential to protect population health and ensure the sustainability of immunization efforts. Studies have debunked unfounded myths regarding vaccine safety, and studies have shown that a strong provider recommendation plays an important role in vaccine conversations [33,34]. Acknowledging the concerns of vaccine-hesitant parents and guardians provides an opportunity to foster trust and dialog, moving toward greater vaccine acceptance.

## 2. Possible Solutions: National and State-Level Strategies and Initiatives

The resurgence of vaccine-preventable diseases such as measles, influenza, and COVID-19 highlights the urgency to address this issue. In response, public health organizations, including CDC, WHO, and various national and state immunization coalitions, have implemented targeted strategies to enhance vaccine confidence. These initiatives include developing culturally and linguistically appropriate messages, launching public education campaigns, training healthcare providers, and enacting supportive legislation.

### 2.1. State Immunization Coalitions and Coordinated Efforts

Various state immunization coalitions across the United States serve as essential networks, composed of public health agencies, non-profit organizations, healthcare providers, educational institutions, and community organizations. These coalitions unify immunization messaging, share resources, advocate for effective vaccine policies, and foster community engagement.

#### 2.1.1. Community-Driven Strategies

The use of localized engagement strategies has shown to be more effective than centralized methods, especially in communities where trust in institutions has diminished. Research has demonstrated that collaborations with recognized community leaders and organizations significantly enhance vaccination program results [35].

Numerous regional programs illustrate the effectiveness of this method. For instance, in California, the Vaccinate All 58 Campaign, launched by the California Department of Public Health, formed collaborations with faith-based organizations, local businesses, and community groups to provide culturally relevant communications and create vaccination services in familiar environments [36]. In Arizona, the pandemic response of the Navajo Nation combined tribal leaders and community health representatives with regional coalitions to create focused outreach initiatives for Native American communities [37]. Likewise, New York City’s Community Engagement Alliance Against COVID-19 Disparities introduced targeted initiatives to improve vaccination rates among various demographic groups [38].

These initiatives highlight the essential components of effective vaccination efforts in the form of tailoring to local settings, fostering relationships with community partners, and distributing precise health information via culturally relevant methods.

#### 2.1.2. Community Outreach and Creative Engagement

Effective outreach efforts combine education with meaningful, community-centered engagement to normalize vaccination and build lasting trust. Coalitions often participate in local events, such as fairs and health expos, providing opportunities for face-to-face interactions that address concerns, dispel misinformation, and encourage vaccination. Examples of creative initiatives in various states include “Pop Up vaccine clinics” by Immunize Colorado at community events like farmers’ markets; the “Vaccine Carnival” initiative hosted by the Texas Rural Health Association; Iowa Immunizes Coalition efforts to deliver vaccination services and education; and “Mobile health van initiative” for vaccination in rural New Mexico [39,40,41,42]. These creative and localized approaches demonstrate how public health initiatives can adapt to meet the needs of diverse communities.

### 2.2. Healthcare Provider Training and Support

It is crucial that healthcare providers are equipped with the skills and resources to address vaccine hesitancy effectively. To this end, the CDC and American Academy of Pediatrics, immunization coalitions, and public health organizations invest in training programs that enhance providers’ communication techniques and empower them to engage with families confidently and compassionately. For instance, American Academy of Pediatrics (AAP) Training Programs focused on improving pediatrician communication skills and incorporated role-playing scenarios to practice addressing vaccine hesitancy with parents [43]. By empowering pediatricians and other frontline healthcare providers, these initiatives ensure that every patient interaction becomes an opportunity to advocate for immunization and public health.

### 2.3. Policy and Legislative Measures

While education and community engagement are essential, policy measures are critical for ensuring high vaccination rates, especially in the face of increasing vaccine hesitancy. Policy measures serve as a critical backbone for maintaining public health and reducing the risk of outbreaks. By combining strict vaccination mandates with educational requirements and incentives, these policies ensure higher immunization rates, build public confidence, and protect vulnerable populations. Some examples from across the United States are presented below.

#### 2.3.1. Mandatory Vaccination Laws

California’s SB 277 law, passed in 2015, eliminated personal belief exemptions for school vaccinations following a significant measles outbreak in 2014. This landmark legislation, supported by public health officials and the state’s immunization coalition, demonstrated the effectiveness of stringent vaccination policies in reducing disease outbreaks [44]. In response to a major measles outbreak, in New York in 2019, religious exemptions for school vaccinations were eliminated, making it one of the strictest states for vaccine requirements. This action led to increased vaccination rates and helped curb the outbreak [45,46]. Maine in 2019 passed legislation to remove both religious and philosophical exemptions for school-required vaccines, further narrowing avenues for nonmedical exemptions [47]. Other states like West Virginia and Mississippi have long-standing laws allowing only medical exemptions for school vaccinations, resulting in some of the highest childhood immunization rates in the United States [48].

#### 2.3.2. Vaccination Mandates for Healthcare Workers

To protect vulnerable populations, several states in the U.S. have implemented vaccination mandates for healthcare workers, like Washington state enforced COVID-19 vaccine mandates for healthcare workers during the pandemic, ensuring the safety of both patients and staff in clinical settings [49].

#### 2.3.3. Policies Supporting Immunization Infrastructure

Several policies aim to strengthen immunization systems and access, for instance, Oregon state expanded access to school-based health centers to improve vaccine delivery in underserved areas [50] and Colorado passed legislation to streamline vaccination exemption processes, requiring parents seeking nonmedical exemptions to complete educational modules about vaccine risks and benefits [51].

### 2.4. Proposed Strategies to Address Cultural, Economic, and Logistical Barriers to Vaccination in the U.S.

To overcome cultural, economic, and logistical barriers to vaccination in the United States and ensure the long-term sustainability of immunization efforts, a comprehensive approach is necessary. To achieve this, data-driven monitoring should be implemented to continuously track vaccination rates, identifying areas of concern to adjust strategies as needed. This ongoing assessment will help keep initiatives aligned with evolving needs. Capacity building is another critical aspect. Healthcare providers and community members should receive training on effective vaccination delivery and outreach practices, equipping them with the skills and knowledge to engage with their communities and improve vaccine uptake. Additionally, advocating for policies that promote vaccination access and uptake will help institutionalize support for these efforts and ensure their sustainability over time.

To address cultural barriers to vaccination, key strategies include fostering community engagement and implementing culturally competent outreach programs as noted above [35,36,37,38,39,40,41,42]. These measures should be customized to meet the diverse and unique needs of communities and populations across the nation.

Additionally, addressing socioeconomic disparities is crucial, as vaccine hesitancy and access barriers disproportionately affect marginalized populations [52]. To overcome these obstacles, it is essential to involve communities in the process by empowering them to design and implement vaccination strategies that are culturally relevant and meet their specific needs. This community-led approach ensures that solutions are both impactful and sustainable. Economic barriers and financial difficulties can be addressed by supporting the provision of immunization at low or no cost through the federally funded vaccines for children (VFC) program [31].

Inadequate access to vaccination sites is an obstacle, particularly for those who reside in remote areas [52]. Offering transportation support and flexible healthcare appointment options, such as extended hours, weekend openings, or walk-in services, would be helpful in overcoming access challenges. Additionally, partnering with healthcare providers to ensure the integration of vaccination with health visits in remote areas and the provision of mobile vaccination clinics [52] that can travel to underserved areas could improve vaccine access. Thus, to address logistical barriers, efforts to bring vaccinations closer to people through practical, community-centered solutions could be helpful. Community-based vaccination locations, such as schools, churches, and recreation centers, provide familiar and welcoming spaces for care. Key considerations in these efforts include understanding geographic variations, such as the unique challenges faced by urban, rural, and tribal communities. Tailoring approaches to the specific needs of these regions is essential for maximizing reach and effectiveness.

### 2.5. Reflections on Challenges

There are challenges; however, these aforementioned strategies might not consistently be effective in every context. Collaborations involving multiple stakeholders, for instance, require considerable time, funding, and staff to operate successfully. In smaller communities, insufficient infrastructure or lack of financial resources can hinder the creation and maintenance of these partnerships. Likewise, the training of community leaders and ensuring their active involvement frequently adds extra pressure on public health systems that are already overwhelmed. The public health system in the U.S. confronts ongoing issues, including obsolete information systems and a shortage of qualified personnel. These shortcomings obstruct the system’s capability to strategize, provide, and enhance services efficiently and effectively. Cultural factors add another dimension of complexity, frequently resulting in less favorable results. A universal method of cultural sensitivity often does not consider the complexities and distinct traits of specific communities. Inconsistent communication presents another major obstacle. Throughout the COVID-19 pandemic, for example, conflicting messages—especially from government representatives—harmed public trust, frequently led to confusion, and decreased compliance with public health guidelines [53].

## 3. Addressing Vaccine Hesitancy with Motivational Interviewing

One of the most effective approaches during provider–patient interactions to deal with vaccine hesitancy is Motivational Interviewing (MI), a communication technique centered on the patient that aims to address ambivalence and encourage intrinsic motivation to alter behaviors [54,55,56].

Motivational Interviewing is a proven, patient-centered communication technique that fosters trust and supports informed decision-making [5].

Pediatricians and providers are uniquely positioned to address vaccine hesitancy due to their frequent and ongoing interactions with families, often starting in infancy and continuing through adolescence. Local pediatric providers in freestanding clinics play a critical role in delivering primary healthcare, constituting approximately 75% of primary care for children in the United States [57]. Furthermore, pediatric clinics often serve as the first point of contact for vaccine education, emphasizing their central role in addressing hesitancy. Providers in these settings are well-placed to understand the cultural and community-specific barriers that influence vaccine decision-making, allowing them to tailor their approach to meet the needs of individual families. The long-standing relationships pediatric providers build with families foster trust, enabling open and honest communication about health concerns, including vaccines. Parents are more likely to discuss fears, misconceptions, and questions about immunizations in a familiar and supportive environment. This trust is pivotal in helping families make informed decisions about vaccination, reducing hesitancy, and improving uptake.

Motivational Interviewing is a communication approach that can help alleviate concerns about vaccinations and provide pediatricians and clinicians with the skills needed to engage patients and parents/guardians through non-judgmental conversation, avoiding confrontation and resistance. Nurses are an integral part of the healthcare teams in the U.S. and play a vital role in supporting providers during conversations about vaccine hesitancy. Their frequent, direct interactions with patients put them in a unique position to establish trust and model the spirit of MI by fostering empathy, collaboration, and patient autonomy. By reinforcing the principles of MI and working closely with providers, nurses help create a supportive environment that empowers patients and families to make informed decisions about vaccination.

MI as an effective tool to counter vaccine hesitancy will be discussed in detail in this section.

### 3.1. Principles of MI

The following section addresses the fundamental principles of MI, its implementation in healthcare, and its vital role in reducing vaccine hesitancy. Particular focus is given to classic MI techniques, including open-ended questions, giving affirmations, reflecting on concerns, requesting permission to share information, and maintaining autonomy throughout the decision-making process. A concise, adaptable framework for implementing MI in clinical settings to address vaccine hesitancy is presented in Box 1 [5,6,55,56]. Unlike conventional methods that depend on persuasion or confrontation, MI emphasizes cooperation, empathy, and respect for the autonomy of patients. By promoting open and judgment-free discussions, MI assists individuals in examining their concerns, allowing them to arrive at informed decisions based on their personal values and priorities. Research shows that MI is highly successful in facilitating health behavior changes, including increased acceptance of vaccinations, by diminishing resistance and enhancing trust [6].

Box 1 **Stepwise guide to implementing Motivational Interviewing (MI) in addressing vaccine hesitancy.**

1. Open-Ended Questions
  o Facilitate meaningful dialog by encouraging patients to share their concerns
  o Example: “What are your main thoughts about this vaccine for your child”?
2. Affirmations
  o Acknowledge and validate patient/parent efforts to make informed choices
  o Example: “I see how much you care about your child’s health, and I appreciate your thoughtfulness”.
3. Reflective Listening
  o Reflect concerns to show empathy and clarify patient/parent perspectives
  o Example: “It sounds like you’re unsure about the long-term safety of the vaccine”.
4. Permission to Share Information
  o Respect autonomy by asking permission before providing evidence-based information
  o Example: “Would it be helpful if I shared how the vaccine was tested for safety”?
5. Empowering Decision-Making by autonomy to parents
  o Reinforce autonomy and support patient confidence in their ability to decide
  o Example: “This is your decision, and I’m here to provide any information you need”.

#### 3.1.1. Asking Open-Ended Questions

A foundational element of Motivational Interviewing (MI) is asking open-ended questions—an approach that encourages patients to share their concerns freely and deeply, rather than restricting responses to “yes” or “no” answers. This technique is particularly effective when engaging with parents who are ambivalent about vaccinating their children. By fostering a safe and non-judgmental space, healthcare providers can uncover the underlying beliefs, fears, and misconceptions that fuel hesitancy, paving the way for meaningful and supportive conversations [5].

For instance, clinicians can shift away from binary questions, such as, “Are you worried about the vaccine”? Instead, they can ask more open-ended and exploratory questions, such as, “What are your main concerns about your teenager getting vaccinated”? or “What have you heard about the vaccine that worries you”? These questions allow parents to articulate their thoughts fully, whether those involve fears about side effects, distrust of the vaccine development process, or misinformation spread by social media [58].

Open-ended questions encourage reflective dialog, which often leads to moments of insight, allowing individuals to reevaluate their concerns in light of accurate information. This method has been shown to foster trust and facilitate behavior change, as it engages patients in a collaborative process rather than imposing solutions on them [55,59].

#### 3.1.2. Offering Affirmations

Once patients share their concerns, offering affirmations becomes a crucial next step. Affirmations are statements that validate and acknowledge patients’ thoughts, feelings, or efforts, reinforcing a sense of respect and understanding. For example, a clinician might say, “It’s clear that you’ve been thinking carefully about what’s best for your child, and I appreciate how much you care about their health”. This approach demonstrates empathy, encourages further dialog, and helps establish rapport [55].

Affirmations are particularly effective in reducing defensiveness, which can otherwise arise when patients feel judged or dismissed. By focusing on the parent’s positive intentions, this technique enhances the emotional climate of the conversation and creates a space for constructive, collaborative engagement [60]. Studies proved that affirmations, when used alongside reflective listening, significantly increase the effectiveness of MI in addressing vaccine hesitancy [6].

#### 3.1.3. Reflecting Concerns

Another core MI technique is reflective listening, which involves paraphrasing or restating patients’ concerns to demonstrate understanding and empathy. For instance, a clinician might respond to a parent’s hesitation about vaccine safety by saying, “It sounds like you’re worried that the vaccine could have long-term side effects that haven’t been studied yet. Is that correct”? This reflective approach validates the parent’s feelings while also encouraging them to further explore their concerns [5].

Reflective listening serves two key purposes: It reassures patients that their concerns are being heard and allows clinicians to gently address misconceptions with accurate, evidence-based information. For example, in response to the above concern, the clinician might continue, “That’s a very understandable worry. Can I share some information about how vaccines are tested and monitored for long-term safety”? Research indicates that reflective listening improves patient–provider communication and helps reduce resistance to change, making it a critical tool in addressing vaccine hesitancy [61].

#### 3.1.4. Seeking Permission to Share Information

A hallmark of MI is respecting patient autonomy, even when providing education. Rather than countering misconceptions directly or offering unsolicited advice, MI practitioners first seek permission to share information. For example, a clinician might ask, “Would it be okay if I shared some information about how the vaccine was developed and tested”? This technique ensures that patients feel in control of the conversation, fostering a sense of collaboration rather than coercion [6,62,63].

Studies suggest that this respectful exchange increases receptiveness to new information, especially among individuals who are initially skeptical or mistrustful of authority figures. Once permission is granted, clinicians can provide tailored, evidence-based information to address the patient’s specific concerns [64]. This collaborative approach aligns with findings from communication research that emphasize the importance of empowering patients in decision-making processes [65].

#### 3.1.5. Giving Autonomy to Parents

Giving autonomy to parents is a core principle of Motivational Interviewing and an essential part of building trust in any healthcare conversation [55]. By allowing parents to feel that they control decisions affecting their teenager’s health, the clinician respects their role as caregivers and acknowledges their right to make informed choices for their family. This approach helps parents feel more comfortable and confident in the decisions they make, knowing they have support but are in control of the final choice. For instance, the clinician may say, “I’ve shared a lot of information, and I want you to know that whatever decision you make is entirely up to you. I’m here to support you in any way you need, and if you have more questions later, just let me know”.

When talking about vaccines, giving autonomy means reminding parents that while you are there to provide guidance and expert advice, the decision is theirs to make. This respectful approach can make a big difference, especially when parents are feeling uncertain or overwhelmed. It helps them see that the clinician is working with them, not pushing them, and that their values and concerns matter.

The process of MI with specific examples of all components is presented in Box 1 [5,6,55,56].

### 3.2. Impact of MI

Motivational Interviewing leverages empathy, trust, and collaboration to address vaccine hesitancy. By validating concerns and empowering patients to make informed decisions, MI strengthens the provider–patient relationship and increases vaccine acceptance. MI enables providers to build better rapport with families, address specific concerns, and navigate challenging conversations. Its structured yet flexible approach ensures respectful, effective communication that supports individual and community health outcomes [6,66].

As presented in Box 1, the concise MI guide is designed for application in clinical research and practice to foster trust, engage patients, and improve vaccination uptake.

Motivational Interviewing (MI) has proven to be a highly effective approach for addressing vaccine hesitancy, with several key studies highlighting its impact. These studies show how MI can help build trust, address concerns, and increase vaccine acceptance, especially when applied thoughtfully in clinical settings. Below, we summarize the findings of some of the most significant research on MI and vaccine hesitancy and explain how our study builds on their work. One important study by Gagneur et al. [6] looked at how MI could be used during postnatal counseling sessions with vaccine-hesitant parents in maternity wards in Quebec, Canada. By engaging parents at a critical moment—right after childbirth—MI-based conversations addressed their concerns and encouraged them to vaccinate their infants. The findings clearly showed that parents who participated in Motivational Interviewing (MI) sessions were significantly more likely to vaccinate their infants than those who did not. This research highlights the effectiveness of MI at critical healthcare touchpoints. Our study extends this by examining how MI techniques can be tailored to other contexts, such as pre-vaccination discussions during pediatric visits or conversations with vaccine-hesitant adults. 

Similarly, a study by Henrikson et al. [65] explored training healthcare providers in communication strategies, including MI, to address parental vaccine hesitancy. The study revealed that clinicians trained in MI had more positive and trusting interactions with parents, which helped reduce resistance. This underscores the value of equipping healthcare professionals with effective communication tools. Our work builds on these findings by emphasizing the importance of practical training programs to help clinicians seamlessly integrate MI principles into their daily practice.

Dube et al. [62] conducted a comprehensive analysis of ways to address vaccine hesitancy. Their findings highlighted Motivational Interviewing (MI) as particularly effective, primarily because it emphasizes listening to patients and avoiding confrontation, which helps build trust and encourage meaningful dialogue. While the analysis confirmed MI’s value, it also noted practical limitations, particularly the time constraints faced in active medical settings. Building on these insights, our review explores methods to adapt MI for busy healthcare environments and suggests combining it with complementary approaches to enhance its practical application.

Opel et al. [67] found that Motivational Interviewing (MI) is particularly effective in pediatric care when physicians validate parents’ concerns without pressuring them to make specific decisions. When healthcare providers displayed empathy and maintained a neutral stance, parents were more open to discussions about vaccinations. Building on this research, we explore the application of MI beyond pediatric care, focusing on its potential for promoting adult vaccinations, including those for influenza and COVID-19. While prior studies confirm MI’s efficacy, healthcare providers often lack clear guidance on its practical application. Our review fills this gap by offering detailed strategies for implementing MI across diverse healthcare settings and patient populations. Additionally, we address common challenges in MI, such as time constraints, and propose solutions to integrate them seamlessly into routine practice. Our aim is to develop a comprehensive approach to vaccine hesitancy by combining MI with broader public health initiatives. 

Table 1 [5,6,59,62,64,65,67] compares MI to other communication models commonly used in vaccine discussions and illustrates the key differences between Motivational Interviewing (MI) and other common communication approaches used to address vaccine hesitancy. This table [5,6,59,62,64,65,67] highlights the unique strengths of MI compared to directive, persuasion-based, and shared decision-making approaches. 

While numerous studies have explored vaccine hesitancy and the use of MI as a communication tool [68,69], our review distinguishes itself by focusing on the practical application of MI techniques in clinical settings. This review focuses on the implementation of Motivational Interviewing techniques for vaccine hesitancy and provides a detailed step-by-step framework that clinicians can use in real-world settings.

Specifically, the review aims to bridge the gap between theory and practice. There is a lack of studies providing guidance on practical, clinician-focused tools for implementation. Our study goes beyond describing MI’s effectiveness to provide a step-by-step framework for real-world application. We include specific examples, such as handling emotionally charged conversations with parents of teenagers, addressing misinformation spread via social media, and navigating scenarios with vaccine-hesitant adults.

Also, the review incorporates broader systemic and contextual factors, and we also explore how MI can be integrated with broader public health strategies, such as addressing the role of social media, combating hyperpolarized misinformation, and empowering healthcare providers through training. This dual focus—interpersonal MI techniques and systemic strategies—offers a more comprehensive approach.

The review offers an approach to customizing MI for vaccine hesitancy specifically. Our study delves into how MI techniques can be tailored to the unique context of vaccine hesitancy. This includes addressing specific psychological barriers, such as fear of side effects or distrust of healthcare systems, which are not universally covered in other MI-related research. In addition, this review provides critical reflections on existing strategies and offers a critical evaluation of where MI and other strategies succeed or fall short, offering insights into how MI can be improved or combined with other communication frameworks to maximize effectiveness.

## 4. Conclusions

Vaccine hesitancy, the reluctance or refusal to vaccinate despite the availability of vaccines, remains a growing concern worldwide. The issue of pediatric vaccine hesitancy in the United States is complex and influenced by a combination of social, cultural, psychological, and systemic factors. Addressing vaccine hesitancy is essential to prevent the re-emergence of vaccine-preventable diseases and requires a multifaceted approach that combines education, community engagement, provider support, and strong policy measures. Motivational Interviewing is an example of a patient-centered communication approach that can help alleviate concerns about vaccinations and equip clinicians with the skills needed to engage patients and parents/guardians through empathy and collaboration, avoiding confrontation and resistance.

At the heart of these efforts lies the critical need to build and maintain trust—trust in vaccines, healthcare providers, and the systems that protect public health. Parents, as primary decision-makers for their children’s well-being, expect clear, compassionate, and evidence-based guidance from healthcare providers. Meeting these expectations fosters confidence and empowers families to make informed decisions that safeguard not only their own children but also the broader community. By prioritizing trust, collaboration, and tailored strategies, we can bridge the gaps of hesitancy and ensure a healthier, more resilient future for all.

## Figures and Tables

**Table 1:** Key differences between Motivational Interviewing (MI) and other common communication approaches.

Aspect	Motivational Interviewing (MI)	Shared Decision-Making	Persuasion-Based Approach	Directive Approach
Communication style	Collaborative, patient-centered. Focuses on assisting patients through their conflicting feelings toward vaccines.	Collaborative, primarily focused on presenting options and allowing the patient to decide.	Argumentative or fact-heavy. Focuses on convincing patients using logic and evidence.	Authoritative or instructional. Provides direct advice without exploring patient issues or concerns.
Focus	Explores the patient or parent’s values, emotions, and personal motivations related to health decisions.	Focuses on presenting all treatment or vaccine options without delving into emotional barriers.	Focuses on overcoming resistance through information and evidence-based persuasion.	Focuses on delivering solutions and recommendations without an in-depth exploration of underlying concerns.
Response to hesitancy	Validates issues and concerns through empathy and reflective listening. Builds trust and reduces defensiveness.	May acknowledge hesitancy but does not necessarily explore underlying emotional or psychological factors.	Often assumes resistance is due to a lack of knowledge and counters it with facts.	May dismiss or downplay concerns. Often focuses on giving directives or “correcting” misconceptions.
Handling misinformation	Encourages patients to discuss their beliefs and sources of information. Gently offers corrections when invited.	Provides evidence-based responses but does not focus heavily on the psychological root of misinformation.	Counters misinformation with evidence but may risk appearing dismissive of patient concerns.	Corrects misinformation directly, potentially leading to defensiveness or mistrust.
Engagement technique	Uses open-ended questions, affirmations, and reflective statements to engage patients in meaningful dialogue.	Encourages shared decision-making but may lack techniques to explore personal doubts or ambivalence.	May engage in debate or heavily focus on providing counterarguments to hesitancy.	Relies on direct statements and recommendations, limiting dialog.
Autonomy of the patient	Respects and emphasizes the patient’s autonomy, allowing them to make their own informed decisions.	Respects patient autonomy by presenting choices but may not address emotional hesitations effectively.	Can feel like pressure to agree with the clinician’s perspective, reducing perceived autonomy.	Tends to minimize autonomy by emphasizing expert authority and clear directives.
Key strengths	Builds trust, reduces defensiveness, and empowers patients to resolve their ambivalence.	Balances autonomy and collaboration, effective for patients ready to make a decision without hesitation.	Effective for patients who respond well to facts and evidence.	Clear and concise communication, which can be effective for patients who prefer direct guidance.
Limitations	Requires time and skilled training to implement effectively.	Does not always address deeper emotional or psychological barriers to vaccine acceptance.	Can backfire if patients feel overwhelmed, judged, or resistant to a persuasion-heavy approach.	May alienate patients by appearing dismissive of their concerns or limiting dialog.
Example dialogs	“May I share what we know about vaccine safety”?	“Here are the options: you can get the vaccine today or revisit the decision at a later date”.	“The data clearly shows vaccines are effective and save lives, and here’s why your concerns are not supported by evidence”.	“The vaccine is safe, and I recommend that you schedule it today”.
